# Circadian control of histone turnover during cardiac development and growth

**DOI:** 10.1016/j.jbc.2024.107434

**Published:** 2024-06-01

**Authors:** Adrian Arrieta, Douglas J. Chapski, Anna Reese, Todd H. Kimball, Kunhua Song, Manuel Rosa-Garrido, Thomas M. Vondriska

**Affiliations:** 1 Department of Anesthesiology & Perioperative Medicine, David Geffen School of Medicine at UCLA, UCLA, Los Angeles, California, USA; 2 Internal Medicine, Heart Institute, Center for Regenerative Medicine, University of South Florida, Tampa, Florida, USA; 3 Department of Biomedical Engineering, School of Medicine and School of Engineering, University of Alabama at Birmingham, Birmingham, Alabama, USA; 4 Division of Cardiology, Department of Medicine, UCLA, Los Angeles, California, USA; 5 Department of Physiology, UCLA, Los Angeles, California, USA; 6 Molecular Biology Institute, UCLA, Los Angeles, California, USA

**Keywords:** chromatin, proteostasis, histone, nucleosome, myocyte

## Abstract

During postnatal cardiac hypertrophy, cardiomyocytes undergo mitotic exit, relying on DNA replication-independent mechanisms of histone turnover to maintain chromatin organization and gene transcription. In other tissues, circadian oscillations in nucleosome occupancy influence clock-controlled gene expression, suggesting a role for the circadian clock in temporal control of histone turnover and coordinated cardiomyocyte gene expression. We sought to elucidate roles for the master circadian transcription factor, Bmal1, in histone turnover, chromatin organization, and myocyte-specific gene expression and cell growth in the neonatal period. Bmal1 knockdown in neonatal rat ventricular myocytes decreased myocyte size, total cellular protein synthesis, and transcription of the fetal hypertrophic gene *Nppb* after treatment with serum or the α-adrenergic agonist phenylephrine. Depletion of Bmal1 decreased the expression of clock-controlled genes *Per2* and *Tcap*, as well as *Sik1*, a Bmal1 target upregulated in adult *versus* embryonic hearts. Bmal1 knockdown impaired *Per2* and *Sik1* promoter accessibility as measured by micrococcal nuclease-quantitative PCR and impaired histone turnover as measured by metabolic labeling of acid-soluble chromatin fractions. Sik1 knockdown in turn decreased myocyte size, while simultaneously inhibiting natriuretic peptide B transcription and activating Per2 transcription. Linking these changes to chromatin remodeling, depletion of the replication-independent histone variant H3.3a inhibited myocyte hypertrophy and prevented phenylephrine-induced changes in clock-controlled gene transcription. Bmal1 is required for neonatal myocyte growth, replication-independent histone turnover, and chromatin organization at the *Sik1* promoter. *Sik1* represents a novel clock-controlled gene that coordinates myocyte growth with hypertrophic and clock-controlled gene transcription. Replication-independent histone turnover is required for transcriptional remodeling of clock-controlled genes in cardiac myocytes in response to growth stimuli.

During neonatal cardiac development in mammals, cardiomyocytes integrate environmental cues such as changes in blood pressure, blood oxygen concentration, and circulating hormones with genetic programming to execute cardiac maturation (1). Part of this process includes a switch from cardiomyocyte proliferation to hypertrophy, which is accompanied by a loss of regenerative capacity within the first week after birth (2). Gene expression has been extensively studied during this period of cardiac maturation, as have the roles of transcription factors and epigenetic modifiers in specifying myocyte cell fate. Time-dependent control of chromatin organization during cardiac maturation is poorly understood. In this study, we sought to understand the role of histone turnover in myocyte growth during the neonatal period.

Since myocytes exit the cell cycle, they must switch from DNA replication-dependent to replication-independent replacement of nucleosomes (the basic packaging units of chromatin). Much evidence suggests that histone replacement is a precise phenomenon outside of the context of replication and that the process of histone replacement is coupled with transcription (3, 4, 5). In the adult heart, transcriptional responses to pathologic growth following pressure overload become entrained in chromatin, such that locus-specific remodeling precedes later transcriptional adaptation (6). Also in adults, nucleosome turnover (as measured by GFP-tagged histone H2B) is higher at actively transcribed genes, including those involved in cell identity and cardiac contractility (3). This study also observed increased turnover at areas of active chromatin as indicated by histone marks such as H3 lysine 27 acetylation (3). However, the role of temporally precise histone and nucleosome turnover in early myocyte development has never been explored.

Circadian clocks function to control rhythmic behaviors of multicellular organisms (7) and these rhythms can be influenced by external time cues, otherwise known as “zeitgebers.” The central clock housed in the suprachiasmatic nucleus in the brain is entrained by light (8), while the peripheral clock in the heart responds to feeding-fasting cycles and catecholamines (9, 10, 11). The molecular clock operates at the cell and organ level, underpinned by a subcellular network of transcriptional and protein circuits originally identified at the molecular level in flies with the discovery of the gene *Period* (12). Clock and Bmal1 are basic helix-loop-helix transcription factors that bind to E-box regulatory elements to promote transcription of a set of clock-controlled genes, including cryptochrome (Cry1/Cry2) and period (Per1/Per2/Per3). Cry and Per form heteromultimers with casein kinase in the cytosol, inducing nuclear translocation, where the complex binds to Clock-Bmal1 heterodimers to repress their own transcription in a negative feedback loop (13). In addition to transcriptional cycling, many intermediate steps controlling protein abundance have been shown to oscillate, including splicing, mRNA degradation, translation, and protein degradation (14, 15). Circadian rhythms participate in normal cardiac development and disease *in vivo*: altered circadian biology is associated with cardiac arrhythmias, myocardial infarction, hypertension, and diabetes (16, 17), and the loss of Bmal1 in myocytes from birth causes dilated cardiomyopathy and metabolic dysfunction (18). Germline deletion of Bmal1 results in dilated cardiomyopathy developing early in life (19), whereas inducible depletion of Bmal1 in cardiomyocytes of adult mice did not affect heart size or function, but did accentuate hypertrophy following hypertrophic stress by pressure overload, angiotensin II or phenylephrine (PE) (20). In noncardiac tissues, Bmal1 has been implicated in daily oscillations of nucleosome occupancy, histone H3 acetylation, and RNA polymerase II (RNAPII) recruitment (21, 22, 23).

In the present study, we sought to examine the role of Bmal1 in cell growth and chromatin remodeling in cultured neonatal rat ventricular myocytes (NRVMs) in response to the established myocyte hypertrophic stimuli serum or PE (24, 25, 26). Serum has been shown to induce clock-controlled gene expression (11); however, the specific factors in serum responsible for this effect are unknown. Since norepinephrine is a component of serum and an adrenergic receptor agonist that also induces clock gene oscillation and myocyte hypertrophy (11), we therefore sought to interrogate if PE, an α-adrenergic receptor agonist, can drive oscillatory clock gene expression. Furthermore, use of cultured cells allows precise temporal control over the levels of hypertrophic signaling caused by serum treatment or α-adrenergic stimulation in the absence of hemodynamic influence or other circulating factors that can influence myocyte growth (9, 27, 28). Finally, this approach allowed precise temporal control of Bmal1 depletion and subsequent evaluation of phenotype in the absence of cell or organ level compensation. We find that Bmal1 is critical for normal myocyte growth and histone turnover, thereby linking circadian control of chromatin remodeling to normal cardiac maturation after birth.

## Results

### Depletion of Bmal1 impairs myocyte hypertrophy and gene expression

**Figure 1:**
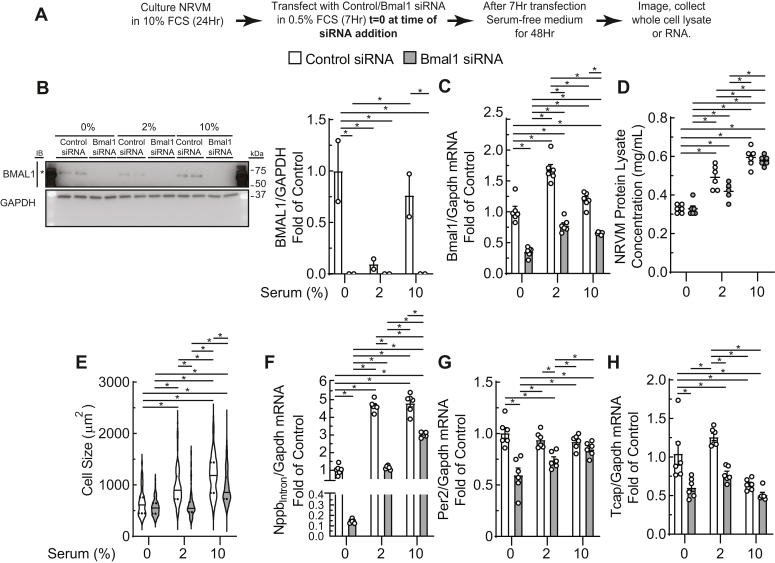
**Bmal1 depletion impairs expression of clock-controlled and hypertrophic genes.***A*, experimental timeline of cultured NRVMs transfected with scrambled or Bmal1-targeted siRNA and treated with increasing serum concentrations. *B* and *C*, Bmal1 and GAPDH immunoblots and RT-qPCR demonstrating Bmal1 knockdown. *D* and *E*, NRVM lysate protein concentration and cell size measurements. *F*–*H*, Nppb_Intron_, Per2, and Tcap RT-qPCR. ∗ indicates significant difference between pairwise comparisons, *p* < 0.05 by two-way ANOVA with Tukey’s *post hoc* analysis; mean ± SEM. Nppb, natriuretic peptide B; NRVM, neonatal rat ventricular myocyte; RT-qPCR, reverse transcriptase quantitative polymerase chain reaction.

To determine whether Bmal1 is required for myocyte hypertrophy in the absence of hemodynamic influence, NRVMs were transfected with siRNA targeting Bmal1 and then incubated with 0, 2, or 10% FCS for 48 h to assess the dose-dependent effect of serum on myocyte growth (Fig. 1*A*). Cultures treated with 0% FCS were supplemented with insulin, transferrin, and selenium (ITS) to maintain cell viability without stimulating hypertrophy (24, 25). Incubation of cultures with 2% FCS significantly decreased Bmal1 protein and increased Bmal1 transcript levels, but greater concentrations in FCS reversed this effect, indicating circadian regulation of clock gene expression at both the transcript and protein levels in myocytes can be influenced by the presence of factors circulating in blood (Fig. 1, *B* and *C*).

Protein concentration of whole cell lysates increased as a function of serum concentration and was not significantly altered by Bmal1 knockdown (Fig. 1*D*). Under serum-free conditions, Bmal1 knockdown did not significantly affect myocyte size; however, Bmal1 depletion prevented myocyte hypertrophy induced by low and high serum concentrations (Figs. 1*E* and S1, *A* and *B*). These observations indicate that Bmal1 expression is dynamic and required at precise levels to engender a growth response; higher levels of serum (10 *versus* 2%) did not induce more Bmal1 (Fig. 1*C*) but did induce greater cell growth, and Bmal1 was necessary at both serum concentrations for this growth (Fig. 1*E*).

We next assessed the effects of Bmal1 depletion on expression of hypertrophic genes and on other clock-controlled genes. *Nppb* is upregulated in hypertrophic myocytes *in vivo* and in culture. We measured the levels of Nppb transcripts containing an intron (Nppb_Intron_) to more accurately reflect *de novo* transcription of this gene in the early stages of myocyte hypertrophy (14, 29). Under serum-free conditions, Bmal1 knockdown resulted in a 90% decrease in Nppb transcription (Fig. 1*F*). Treatment with 2% or 10% serum induced robust increase in Nppb transcript levels, an effect partially attenuated by depletion of Bmal1 (Fig. 1*F*). Although control transfected cells displayed no further increase in Nppb_Intron_ between 2% and 10% FCS, NRVM lacking Bmal1 showed a continued upregulation of Nppb_Intron_. To examine other clock genes, we focused on Per2, a known Bmal1 target across tissue type (7), and titin-cap (Tcap), an established striated muscle-specific clock-controlled gene (30). Bmal1 knockdown significantly decreased the abundance of Per2 under basal conditions or following 2% FCS, with no change after 10% FCS (Fig. 1*G*). A similar pattern of Tcap expression was observed: Bmal1 knockdown significantly decreased Tcap expression at 0 and 2% FCS, with no significant difference after 10% FCS (Fig. 1*H*). These results suggest that Bmal1 is required for myocyte hypertrophy and the regulation of hypertrophic gene transcription in neonatal myocytes.

### Replication-independent histone H3.3a expression is required for myocyte growth and gene expression

**Figure 2:**
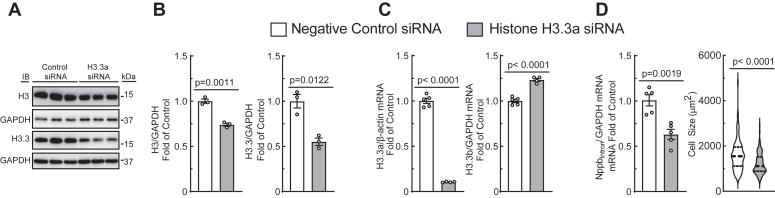
**Replication-independent histone turnover is required for myocyte growth and hypertrophic gene expression.** NRVMs were transfected with siRNA against H3.3a, and knockdown was confirmed by immunoblot (*A* and *B*). H3.3a knockdown results in decreased H3.3a but not H3.3b transcript levels (*C*), and a decrease in Nppb_Intron_ and cell size (*D*). *p* Values from unpaired *t* tests; mean ± SEM. Nppb, natriuretic peptide B; NRVM, neonatal rat ventricular myocyte.

Neonatal myocytes undergo limited cell division in culture (26). Thus, we hypothesized that the majority of histone turnover occurs in a replication-independent manner, likely concomitant with *de novo* gene transcription (4). To determine whether replication-independent histone variants are required for myocyte-specific fetal hypertrophic gene expression, NRVMs were transfected with siRNA targeting the replication-independent histone variant H3.3a, which is exchanged on chromatin during transcription (4, 5). This approach decreased histone H3.3 levels by 50% and total H3 levels by 25% (Fig. 2, *A* and *B*); reverse transcriptase quantitative polymerase chain reaction (RT-qPCR) shows that this depletion is specific for H3.3a, Fig. 2*C*). Knockdown of H3.3a impaired hypertrophic gene transcription (as indicated by Nppb_Intron_ levels) and decreased myocyte size (Fig. 2*D*), consistent with the notion that replication-independent histone H3.3 turnover is critical for *de novo* fetal hypertrophic gene transcription and cell growth in myocytes.

### Bmal1 depletion disrupts histone stoichiometry and impairs histone turnover

**Figure 3:**
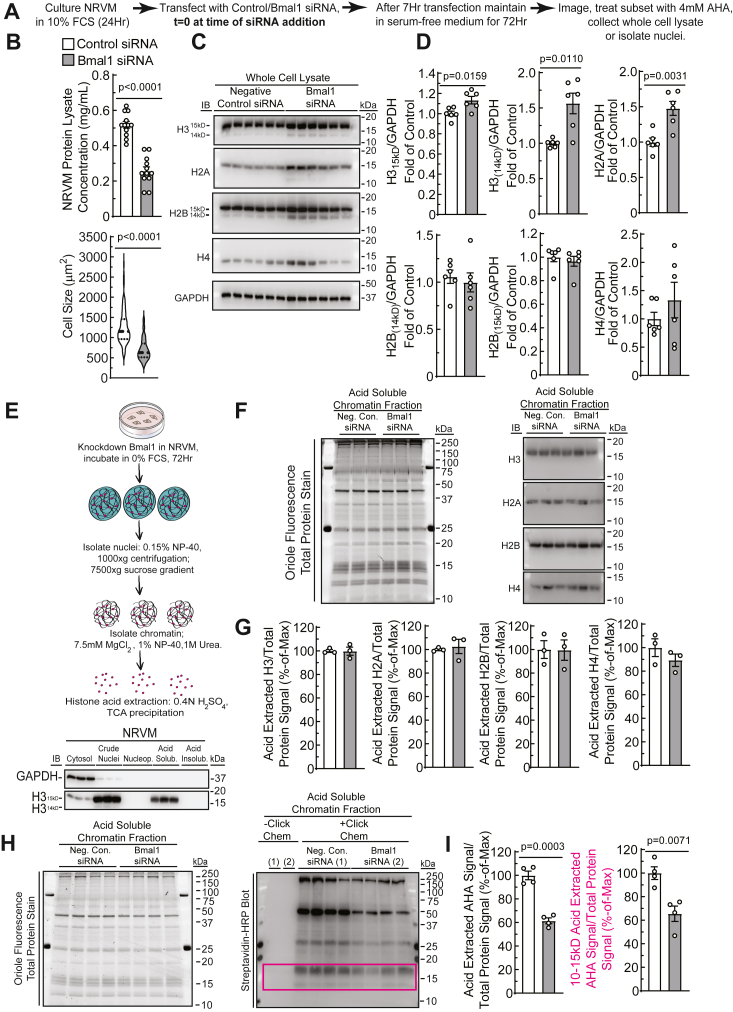
**Bmal1 depletion disrupts histone stoichiometry and impairs histone turnover.***A*, experimental workflow of NRVM transfection and AHA treatment. *B*, protein lysate concentration and cell size measurement of control or Bmal1 depleted cells. *C*, immunoblotting of protein lysates for core histones and GAPDH (quantified in *D*; *p* values from unpaired *t* tests). *E*, diagram of nuclear and chromatin isolation as validated by histone H3 and GAPDH immunoblotting. *F*, oriole fluorescence stain of total protein and histone H3, H4, H2A, and H2B immunoblots of acid-soluble chromatin fractions. Immunoblot signal was normalized to total protein oriole fluorescence signal from control or Bmal1-depleted NRVM (quantitation shown in *G*). *H*, oriole fluorescence staining and streptavidin-HRP blot of acid-soluble chromatin fractions following click-chemistry with biotin-alkyne. *I*, quantitation of total AHA-labeled acid-soluble chromatin fractions, normalized to oriole fluorescence (*p*-values from unpaired *t* tests; mean ± SEM). AHA, L-azidohomoalanine; HRP, horseradish peroxidase; NRVM, neonatal rat ventricular myocyte.

Having linked histone abundance with myocyte growth, we next sought to definitively test whether Bmal1 regulates histone expression and assembly into chromatin. In this experiment, cultures were incubated in serum-free medium for 72 h to minimize the effects of serum-borne factors that can affect clock activity (Fig. 3*A*). We next assessed the relative levels of histone H2A, H2B, H3, and H4, in whole cell lysate and in acid-soluble chromatin fractions relative to GAPDH and total protein, respectively. Depletion of Bmal1 led to a decrease in both total protein levels and cell size (Fig. 3*B*). When controlling for total protein abundance, the levels of H3 and H2A, but that of neither H2B nor H4, were increased after Bmal1 knockdown (Fig. 3, *C* and *D*). We also noted the presence of a lower molecular weight band in our H3 and H2B immunoblots, with significant accumulation of the H3 species after Bmal1 knockdown (H3_14kD_, H2B_14kD_; Fig. 3*D*).

We next sought to distinguish total histone levels from those associated with chromatin in the nucleus, using an established subnuclear fractionation method (31). This approach revealed H3_14kD_ to be associated with intact nuclei, but not with acid-soluble chromatin fractions, suggesting this band reflects H3 that has not been incorporated into chromatin (Fig. 3, *C–F*). Furthermore, we found no differences in histone abundance at the level of chromatin (Fig. 3, *F* and *G*), suggesting that differences in total abundance of histones are maintained in a population not bound to chromatin, *i.e.*, as part of the pool available for nucleosome exchange.

To determine if there is a decrease in histone turnover on chromatin 72 h posttransfection, NRVMs were metabolically labeled with L-azidohomoalanine (AHA) for 4 h (Fig. S2*A*), after which acid-soluble chromatin fractions were isolated and subjected to click chemistry with biotin-alkyne, followed by SDS-PAGE, and streptavidin-horseradish peroxidase (HRP) blotting (32). Consistent with the observed decrease in total cellular protein (Fig. 3*B*) and in AHA incorporation into total protein following Bmal1 knockdown (Fig. S2*B*), streptavidin-HRP blots revealed significantly less metabolic labeling of acid-soluble chromatin fractions with Bmal1 knockdown (Fig. 3, *H* and *I*), including at the molecular weight band where AHA-labeled histones have previously been shown to migrate (32).

**Figure 4:**
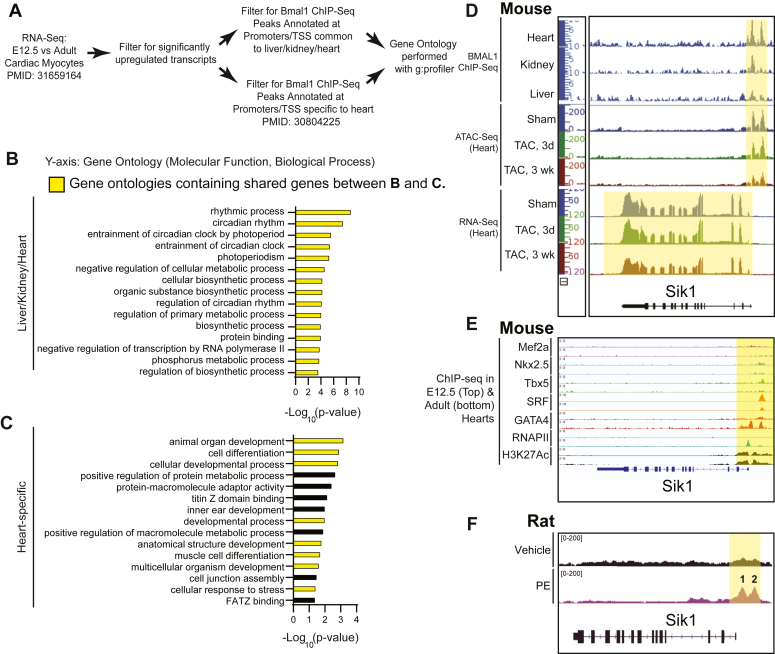
**Sik1 is a novel clock-controlled gene with cardiac-specific chromatin organization that is altered in response to growth stimuli.***A*, diagram of analyses to identify Bmal1 targets upregulated in the adult as compared to the embryonic heart and to identify cardiac-specific Bmal1 candidates. g:Profiler analyses indicating Bmal1-regulated gene ontologies common to the liver, kidney, and heart (*B*), and Bmal1-regulated gene ontologies specific to the heart (*C*). *D*, overlap of Bmal1 ChIP-seq data (21) from indicated mouse tissues and ATAC-seq and RNA-seq data of Sik1 from hearts of mice subjected to transaortic constriction-mediated cardiac hypertrophy (6). *E*, overlay of Mef2A, Nkx2.5, Tbx5, SRF, GATA4, RNAPII, and H3K27Ac ChIP-seq data of Sik1 from embryonic and adult mouse hearts (35). *F*, ATAC-seq data of the Sik1 loci generated from NRVM, demonstrating increased Sik1 chromatin accessibility after treatment with PE (38). ATAC-seq, assay for transposase-accessible chromatin sequencing; ChIP-seq, chromatin immunoprecipitation sequencing; NRVM, neonatal rat ventricular myocytes; PE, phenylephrine; RNAPII, RNA polymerase II; Sik1, salt inducible kinase 1.

Following these observations, we next sought to determine whether these low molecular weight bands were histones, and whether replication-independent histone turnover in NRVM is sensitive to growth stimuli. To this end, serum starved NRVMs were cotreated with 4 mM AHA with or without 50 μM PE following Bmal1 knockdown (Fig. S3*A*). We observed accumulation of newly synthesized proteins (relevant histone bands in 10–15kD boxed region, Fig. S3*B*) following AHA labeling, with minimal change due to PE. As observed in Figure 4, *H* and *I*, the depletion of Bmal1 led to an inhibition of new histone protein synthesis (Fig. S3, *B* and *C*). These histone proteins comigrate with those observed in samples from fibroblasts labeled with 4 mM AHA 10 to 15 h post treatment with 20% FCS (Fig. S4, *A−C*); parallel cultures treated with serum for 15 h revealed a significant increase in cyclin A2, which is required for S-phase (33, 34), and therefore replication-dependent histone turnover (Fig. S4*D*). Of note, we observe that under these experimental conditions, the level of histone labeling in myocytes is ∼10% of what is observed in fibroblasts undergoing replication-dependent histone turnover (*not shown*). There are fewer newly synthesized histones associated with chromatin after Bmal1 depletion (Fig. S3*C*) but the total amount of histone levels on chromatin does not change (Fig. 3, *E−G*): these findings suggest that Bmal1 depletion leads to less histone turnover on chromatin.

### Sik1 is a novel clock-controlled gene induced by growth stimuli

Having shown that Bmal1 acts to remodel chromatin and influence transcription in the postnatal period, we next sought to identify myocyte-specific Bmal1 target gene(s) driving this phenomenon. We examined a transcriptomic study of embryonic and adult mouse myocytes (35) and compared genes significantly upregulated in the adult heart with experimentally determined regions of Bmal1 binding as measured by ChIP-seq (21). When analyzed by g:Profiler, the genes occupied by Bmal1 across organs tended to be enriched in pathways associated with circadian regulation (Fig. 4, *A* and *B*), including rhythmic biological processes, clock entrainment, and photoperiodism. Furthermore, those Bmal1 peaks specific to the heart were enriched for processes related to muscle cell differentiation and development (Fig. 4*C*). We focused on the gene encoding Sik1, because it has been previously shown to mediate myocyte hypertrophy in response to a growth stimulus in the adult heart (36) and also found to contribute to circadian entrainment of the suprachiasmatic nucleus in response to light or serum shock (8), thereby satisfying the criteria of a clock-controlled gene that can influence myocyte growth and entrainment of the myocyte clock. To explore this gene further, we examined Bmal1 ChIP-seq data (6, 21) from the heart, kidney, and liver along with ATAC-seq data from mouse hearts subjected to transverse aortic constriction-induced cardiac hypertrophy (6, 21). This analysis revealed two Bmal1 binding sites, which colocalize with a cardiac-specific pattern of chromatin accessibility at the Sik1 promoter (Fig. 4*D*). RNA-seq data from the same mouse model of pressure overload show decreased levels of Sik1 chromatin accessibility and transcription as a function of time after injury (Fig. 4*D*), consistent with previous reports demonstrating that pressure overload can suppress clock-controlled gene expression (10). Using data from a separate experiment examining Mef2A, Nkx2.5, Tbx5, Srf, Gata4, H3K27Ac, and RNAPII localization in embryonic and adult mouse hearts by ChIP-seq (35), we observed a greater binding of RNAPII and Gata4 in the Sik1 promoter in the adult *versus* embryo (Fig. 4*E*). Because Gata4 transcriptional activity is known to increase after a growth stimulus (37), we examined a published ATAC-Seq dataset from NRVM treated with PE (38)—the same model system used in this study. Data from this study observed two ATAC-seq peaks upstream of the Sik1 promoter (Fig. 4*F*), mimicking the finding from the adult mouse heart (Fig. 4*D*), and confirming cell type-specific, growth stimulus-induced chromatin remodeling at this locus in two separate species (rat and mouse).

### Bmal1 is required for Sik1 promoter accessibility and transcription following growth stimuli

**Figure 5:**
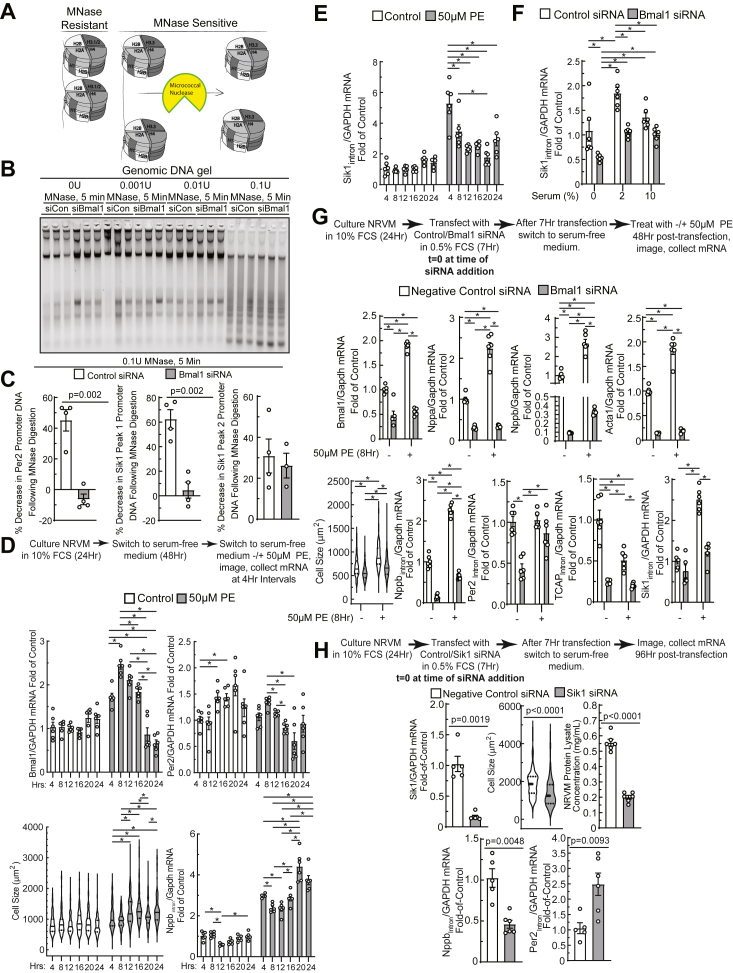
**Bmal1 is required for Sik1 promoter accessibility and transcriptional induction in response to a growth stimulus.***A*, diagram of MNase assay, showing accessibility and degradation of open regions of chromatin. *B*, optimization of MNase for examination of chromatin compaction. In addition, 0.1U for 5 min led to uniform digestions: this global pattern was unaffected by Bmal1 depletion. *C*, change in promoter DNA (indicative of MNase degradation) at Per2 and two Sik1 loci highlighted in Fig. 4*E* and effect of Bmal1 depletion (*p* values from unpaired *t* tests). *D*, experimental workflow of PE treatment. Clock-controlled gene transcripts measured by RT-qPCR and effect of PE on hypertrophy and nascent Nppb transcription (Nppb_Intron_). *E*, effect of PE on nascent Sik1 transcription (Sik1_Intron_) and the role of Bmal1 on this process (*F*). For *D* and *E*, ∗ indicates *p* < 0.05 by one-way ANOVA with Tukey’s *post hoc* analysis for pairwise comparisons in the same treatment condition; for (*F*), ∗ indicates *p* < 0.05 by two-way ANOVA with Tukey’s *post hoc* analysis for pairwise comparisons. *G*, experimental workflow for PE treatment and Bmal1 depletion. Abundance of Bmal1 and fetal gene transcripts (Nppb, Nppa, and Acta1) were assessed by RT-qPCR, and hypertrophy *via* cell size measurements. Transcription of additional clock-controlled gene levels measured by RT-qPCR include Per2_Intron_, Tcap_Intron_, and Sik_Intron_ (∗ indicates *p* < 0.05 by two-way ANOVA with Tukey’s *post hoc* analysis for pairwise comparisons). *H*, experimental workflow for Sik1 depletion. Myocyte hypertrophy was assessed by cell size, Nppb_Intron_ RT-qPCR, and total cellular protein measurements. Per2_Intron_ RT-qPCR performed to examine clock-controlled gene transcription (*p* values from unpaired *t* test; mean ± SEM). PE, phenylephrine; Nppb, natriuretic peptide B; RT-qPCR, reverse transcriptase quantitative polymerase chain reaction; Sik1, salt inducible kinase 1.

We next sought to experimentally confirm these observations in the present study and determine the chromatin remodeling mechanisms underpinning Bmal1-dependent regulation of Sik1 in the setting of neonatal cardiac growth. To test whether these putative regulatory regions in the Sik1 promoter are remodeled in response to a growth stimulus, we used a promoter accessibility assay to directly measure chromatin compaction (39). An MNase dose-response was performed, whereby digestion with 0.1U MNase yielded a DNA laddering pattern indicative of partial digestion that was apparently unaffected by Bmal1 depletion (Fig. 5, *A* and *B*). This result suggests that global chromatin compaction is not affected by Bmal1 depletion.

As a positive control for regulation at the promoter region of specific genes, we tested Per2, a gene known to be regulated in a circadian manner dependent on Bmal1. MNase-qPCR of the resultant DNA revealed that under basal conditions, ∼40% less Per2 promoter DNA was present following MNase digestion. In contrast, Bmal1 knockdown resulted in a protection of the Per2 promoter region (*i.e.* less of a decrease, Fig. 5*C*), indicating that the normal role of Bmal1 is to keep this locus open. We next examined the two peaks of accessibility identified from the prior examination of the Sik1 locus, which we have deemed peak 1 and peak 2 (Fig. 4*F*). We observed a 60% decrease in Sik1 promoter peak 1 under basal conditions and, similar to the Per2 promoter, depletion of Bmal1 prevented the ability of MNase to access this locus (again suggesting the normal role of Bmal1 is to render this region accessible, such that Bmal1 depletion led to less availability to the nuclease; Fig. 5*C*). In contrast, peak 2 in the Sik1 promoter was impervious to the presence of Bmal1 (Fig. 5*C*). In addition to differential regulation of the locus by Bmal1, this observation may be due to differences in DNA base composition of target DNA, which can influence digestion by MNase (40), or differences in other chromatin-binding factors occupying this site.

It has been previously shown that the treatment of isolated cardiac myocytes with the catecholamine norepinephrine drives both myocyte hypertrophy and oscillatory expression of clock-controlled genes (11, 25). Since the expression of clock-controlled genes increases between the embryonic and adult heart, and mammals undergo a postnatal catecholamine surge that is critical for cardiac maturation (41), we assessed whether expression of clock-controlled genes, as well as Sik1_Intron_ and Nppb_Intron_ expression (to measure new transcription), increases in response to α-adrenergic stimulation. NRVMs were treated with the α-adrenergic receptor agonist PE (24) in serum-free medium for 4, 8, 12, 16, 20, and 24 h (Fig. 5*D*). Consistent with the time scale on which Bmal1 has been shown to reach peak expression after norepinephrine treatment (11), Bmal1 transcript levels peaked after 8 h of PE treatment, followed by a gradual reversion to basal levels (Fig. 5*D*). Expression of Per2, an established Bmal1 target, varies over the time course of this experiment in the absence of PE and peaks at 8 h post PE treatment, with a trough of expression appearing at 20 h (Fig. 5*D*). These results support the notion that α-adrenergic stimuli can drive clock-controlled gene expression (for the case of Per2) and alter the phase of their expression (Fig. 5*D*). Along this same time course, PE induced robust hypertrophy and *de novo* transcription of Nppb_Intron_ (Fig. 5*D*). Under control conditions, a trough of Nppb_Intron_ was observed at 12 h, and PE treatment resulted in increased levels of Nppb_Intron_, with oscillation occurring within a 24-h period (Fig. 5*D*). Similar to Bmal1, Sik1 reaches peak expression after 4 to 8 h of PE treatment, followed by a gradual reversion of expression (Fig. 5*E*). Additionally, knockdown of Bmal1 decreased Sik1_Intron_ transcript levels at 0 and 2% FCS, with no significant difference at 10% FCS (Fig. 5*F*). Depletion of Bmal1 also impaired PE-mediated hypertrophy as indicated by cell size measurement and RT-qPCR of fetal genes Nppa, Nppb, and α-skeletal actin (Acta1) (Fig. 5*G*). Furthermore, Bmal1 knockdown impaired basal transcription as well as the PE-induced transcriptional response of Nppb_Intron_, Per2_Intron_, TCAP_Intron_, and Sik1_Intron_ (Fig. 5*G*). We also observed that PE restores transcription of Per2_Intron_ in the Bmal1 depleted condition and results in transcriptional suppression of TCAP_Intron_ (Fig. 5*G*).

Sik1 is required for myocyte hypertrophy in response to a growth stimulus in adult mice (36). We observed that Sik1 depletion alone significantly decreased myocyte size and total protein levels, accompanied by decreased Nppb_Intron_ and increased Per2_Intron_ transcription (Fig. 5*H*), highlighting roles for Sik1 in myocyte hypertrophy and tempering of clock-controlled gene transcription. These results support the notion that Bmal1 is critical for maintaining both accessibility and remodeling of clock-controlled gene transcription (such as Sik1 and TCAP) in response to growth stimuli.

**Figure 6:**
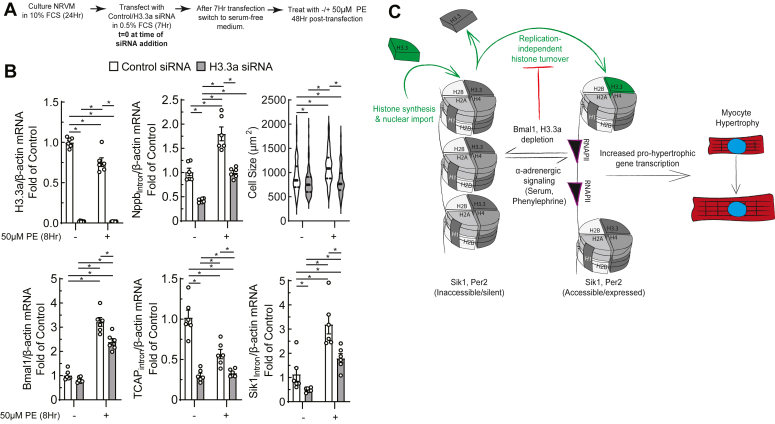
**Replication-independent histone variant H3.3a is required for clock-controlled gene transcription in response to α-adrenergic stimulation.***A*, experimental workflow of PE treatment and Bmal1 depletion. *B*, effect of PE on histone H3.3a as measured by RT-qPCR. Hypertrophy was assessed by Nppb_Intron_ RT-qPCR and cell size. Clock-controlled gene levels were measured by RT-qPCR, including Bmal1, Tcap_Intron_, and Sik1_Intron_ (∗ indicates *p* < 0.05 by two-way ANOVA with Tukey’s *post hoc* analysis for pairwise comparisons; mean ± SEM). *C*, summary diagram: in response to a hypertrophic stimulus, Bmal1 binds transcriptionally inactive and inaccessible clock-controlled target genes and activates their transcription *via* coordination of histone turnover. Sik1 then executes prohypertrophic actions, while Per2 serves to negatively regulate Bmal1 transcriptional activity. Bmal1 depletion impairs this chromatin remodeling cascade. PE, phenylephrine; Nppb, natriuretic peptide B; RT-qPCR, reverse transcriptase quantitative polymerase chain reaction; Sik1, salt inducible kinase 1.

To demonstrate that transcriptional remodeling of clock-controlled genes in response to a growth stimulus requires replication-independent histone turnover, we depleted H3.3a in NRVMs followed by PE treatment to stimulate growth. In addition to decreasing basal cell size (shown also in Fig. 2), H3.3a knockdown impaired Nppb_Intron_ transcription and myocyte growth in response to PE (Fig. 6, *A* and *B*). Furthermore, we observed that H3.3a depletion impaired induction of Bmal1, and phenocopied the effects of Bmal1 knockdown by inhibiting *de novo* transcription of TCAP_Intron_ and Sik1_Intron_.

In summary, our results suggest that the depletion of Bmal1 disrupts histone stoichiometry and impairs histone turnover, resulting in chromatin reorganization and decreased accessibility of the Sik1 and Per2 promoters. These actions impair the transcriptional response of clock-controlled genes following α-adrenergic stimulation and concomitant myocyte hypertrophy. Additionally, we observe that replication-independent histone turnover is required for transcriptional remodeling of clock-controlled genes and myocyte hypertrophy in response to α-adrenergic stimulation.

## Discussion

In this study, we sought to determine whether Bmal1 acts to regulate histone turnover in the setting of neonatal cardiomyocyte growth. Our findings demonstrate that Bmal1 is required for cardiomyocyte hypertrophy in response to serum or α-adrenergic stimulation and that this effect involves chromatin remodeling and transcriptional activation of the salt inducible kinase 1 (Sik1) locus. We chose to examine PE as a growth stimulus because previous investigations have implicated catecholaminergic signaling in circadian regulation of cardiomyocyte function (11). We also examined serum as a growth stimulant, which has hallmarks of physiological growth, but did not examine other known components of serum in isolation, such as insulin-like growth factor or triiodothyronine/T_3_, because neither of these have been implicated in circadian regulation in myocytes, and T_3_ has been shown to act antithetically to PE (42). To our knowledge, whether other pathologic or physiologic hypertrophic agonists regulate Bmal1 and clock gene expression in neonatal myocytes is unknown. To our surprise, higher concentrations of fetal calf serum (FCS) led to a decrease in Bmal1 transcript levels (Fig. 1). Although this experiment does not provide insights into the changes in myocyte growth *in vivo* during the first postnatal days, we speculate that these changes in Bmal1 may reflect the alterations in blood-borne myocyte hypertrophic factors during normal growth. Our findings also indicate that in response to changes in serum concentration, Bmal1 controls transcript abundance of Per2, Sik1, and natriuretic peptide B (Nppb) *via* regulation of *de novo* transcription, through a process that involves histone H3.3a. These results together with our micrococcal nuclease-quantitative PCR (MNase-qPCR) results imply but do not prove that Bmal1 alters the accessibility of these genes to transcriptional machinery in response to changes in the concentration of blood-borne Zeitgebers. An examination of genome-wide effects of Bmal1 on chromatin accessibility would require an approach such as assay for transposase-accessible chromatin sequencing (ATAC-seq) and RNA-seq experiments, combined with focused analyses of locus-specific regulation.

A requirement for replication-independent turnover for myocyte hypertrophy is supported by our observation that depletion of histone H3.3a, a replication-independent histone H3 variant (4, 5), resulted in smaller myocytes, decreased Nppb_Intron_ transcription, and impaired hypertrophy, and impaired transcriptional induction of Sik_Intron_ in response to PE. Interestingly, we observed that PE treatment decreased TCAP_Intron_ transcription  by ∼50%, whereas histone H3.3a knockdown decreased TCAP_Intron_ transcription by ∼75%. These results were phenocopied by Bmal1 knockdown, supporting the notion that clock-controlled gene transcriptional remodeling is reliant on histone H3.3a turnover. Additionally, we characterized Sik1 as a novel clock-controlled gene, exemplified by its oscillatory expression in response to PE treatment, and demonstrated that Sik1 is required for hypertrophic gene transcription, as Sik1 knockdown impairs Nppb_Intron_ transcription and decreases myocyte size and total protein content.

Prior to this study, multiple models of *in vivo* Bmal1 depletion have been reported. One study reports early onset dilated cardiomyopathy characterized by small cardiomyocytes in germline Bmal1 KO mice (19), whereas another study reports unchanged cardiac myocyte size with increased fetal gene expression when Bmal1 is knocked out specifically in cardiac myocytes at E18.5 (18). A third study showed that an inducible depletion of Bmal1 in adult myocytes did not alter the cell size but rendered the mice more susceptible to stress-induced hypertrophy (20). These prior observations demonstrate different roles for Bmal1-mediated gene regulation at different developmental time points, cautioning against extrapolation between effects in the adult and neonate, and providing rationale for examining clock-dependent chromatin remodeling and myocyte growth in the neonatal period in the present study. Our data may also help to reconcile these previous experiments: we show that Bmal1 knockdown in the absence of external hypertrophic stimuli results in smaller cardiomyocytes and impaired Nppb_Intron_ transcription. However, Bmal1 knockdown in myocytes in the presence of external hypertrophic stimuli (2% and 10% FCS) increased Nppb_Intron_ transcription in a serum concentration-dependent manner and impaired myocyte hypertrophy (Fig. 1*F*). The results reported here, along with the previous reports described above, suggest that both timely external hypertrophic cues and a functioning myocyte clock are required for myocyte hypertrophy. A recent study also implicated Bmal1 in miRNA transcriptional regulation in the adult heart (43), meaning it is likely that some of the transcriptome regulation by this pathway involves noncoding RNA. Previous studies have demonstrated that Bmal1 recruitment of cryptochrome proteins Cry1 and Cry2 results in repression of Bmal1 targets (44). Whether this interaction antagonizes transcriptional induction of Sik1 in NRVMs, perhaps *via* antagonization of Gata4, remains to be tested.

It has been previously reported that Bmal1 and Clock act as pioneer transcription factors (23), disrupting DNA-histone contacts to drive nucleosome dynamics and transcription. In the present study, Bmal1 knockdown prevented MNase digestion of Per2 and Sik1 promoter DNA, indicative of higher occupancy or increased stability of nucleosomes at these sites in the absence of Bmal1. Depletion of Bmal1 did not affect total histone levels on chromatin (Fig. 3, *E–G*) but did decrease the association of newly synthesized histones with chromatin, suggesting that Bmal1 orchestrates histone exchange on chromatin (Fig. 6*C*). Further experimentation using labeling of histones, followed by capture and sequencing of nucleosomes containing new histones, will yield spatiotemporal resolution regarding which loci undergo active turnover in the setting of myocyte hypertrophy. Measurement of histone turnover across the genome, in conjunction with chromatin immunoprecipitation sequencing (ChIP-seq) for established histone posttranslational modifications, would also increase our understanding of an epigenetic code dictating histone turnover.

When performing the gene ontology analysis to identify genes regulated by Bmal1 in a cardiac-specific manner, only Sik1 displayed two Bmal1 binding sites in its promoter, suggesting this gene was a promising candidate. Our studies add to the evidence (36) implicating Sik1 as a regulatory node in hypertrophic signaling in cardiac myocytes, revealing a role for Bmal1 in this process. The role of other known cardiac transcription factors in Sik1 regulation is unclear and will require use of genetically engineered animals with tagged versions of these proteins (35) because of low expression and ineffectiveness of available antibodies against endogenous proteins. In addition to our other measurements implicating Sik1 in myocyte growth, we demonstrate that the depletion of Sik1 impairs protein synthesis. Sik1 is a multifunctional signaling kinase, with roles in a wide range of cellular processes ranging from inflammation to metabolism (45). To our knowledge, there is no evidence Sik1 directly phosphorylates, binds to, or regulates protein synthesis or degradation machinery, and thus our observations cannot distinguish its effects on protein synthesis as direct molecular regulation *versus* indirect actions resultant from other signaling to prevent hypertrophy.

In summary, we provide evidence that Bmal1 coordinates histone turnover and mediates chromatin accessibility at genes involved in neonatal myocyte growth. We also demonstrate that Sik1 represents a novel clock-controlled gene newly implicated in both neonatal myocyte hypertrophy and clock entrainment that possesses a unique Bmal1-dependent signature of chromatin organization. This work sets the stage for future studies interrogating how Bmal1 orchestrates nucleosome proteostasis in postnatal cardiac myocytes and resolving the role of replication-dependent and -independent forms of histone turnover.

## Experimental procedures

### Isolation of NRVMs from rat hearts

All animal studies were approved by the UCLA Animal Research Committee in compliance with the National Institutes of Health Guide for the Care and Use of Laboratory Animals. Myocytes were isolated as follows: four litters of neonatal pups (approximately 48 animals, P2-3) were euthanized by decapitation, and atria were removed from excised hearts. Ventricles were then briefly rinsed in ice-cold 1× ADS buffer (116 mM NaCl, 18 mM Hepes, 845 μM NaHPO4, 5.55 mM glucose, 5.37 mM KCl, 831 μM MgSO4, and 0.002% phenol red, pH 7.35 ± 0.5) and minced *via* ∼200 cuts with sterile micro scissors. Minced ventricles were then subjected to 5 to 10 serial digestions in a Wheaton 356945 Celstir 50 ml Jacketed Glass Spinner Flask with Double Sidearms at room temperature (21 °C) using 300 μl/heart of ADS buffer containing 0.1% Trypsin (Sigma-Aldrich, Cat. No. T4799) and 0.002% DNase (Worthington Biochemical Corp, Cat. No LS002006). Each digestion lasted for 10 to 20 min. The cell suspension from the first digestion was discarded as it contained unwanted dead cells and red blood cells. The cell suspension from each subsequent digestion was then combined with FCS to a concentration of 50% FCS. The pooled cell suspensions were then filtered with a 100 μm nylon cell strainer (Foxx Life Sciences, Cat. No. 410-0003-OEM) and then centrifuged for 8 min at 1500*g*. The resulting cell pellet was then resuspended in 8 ml of 1×ADS buffer. Stock Percoll was prepared by combining nine parts of Percoll (cat# 17-0891-02, GE HealthCare) with one part of clear (without phenol red) 10×ADS. The stock Percoll was used to make the Percoll for the top (density= 1.059 g/ml; 1 part Percoll stock added to 1.2 parts 1× ADS without phenol red) and bottom (density= 1.082 g/ml; 1 part Percoll stock added to 0.54 parts 1× ADS with phenol red) layers. The gradient, consisting of 4 ml top Percoll and 3 ml bottom Percoll, was set in a 15 ml conical tube by pipetting the top Percoll first, and layering the bottom Percoll gently underneath. The cells (in 2 ml red 1× ADS buffer) were layered on the top of the discontinuous percoll gradient (4 gradients in total) and centrifuged at 1500*g* for 30 min at 4 °C, with no deceleration break to separate the myocytes from nonmyocytes. The myocytes, concentrated between the two percoll layers as well as myocytes that centrifuged to the bottom of the tube, were then collected, washed once with 1× ADS buffer, and resuspended in plating medium composed of Dulbecco's modified Eagle's medium (DMEM)/F:12 (Thermo Fisher Scientific, Cat. No. 11330-32) supplemented with 10% FCS and penicillin/streptomycin.

### Plating of NRVMs

Fibronectin (Thermo Fisher Scientific, Cat No. 33016015) dissolved at 1 mg/ml in DNase/RNase-free molecular-grade water was further diluted to 5 μg/ml in DMEM/F:12 containing penicillin/streptomycin and then added at 1 ml/well to 6-well dishes (Corning, Cat. No. 3516), which were then incubated at 37 °C for 30 min. The fibronectin solution was aspirated from the dishes, and the Percoll-purified myocytes suspended at 175,000 cells/ml in the plating medium were then cultured on the fibronectin-treated 6-well dishes at 350,000 cells/well, 2 ml of media/well, with each well constituting an independent biological replicate. Cultures were then incubated at 37 °C in an incubator infused with 95% N_2_/5% CO_2_ for 24 h. Cells were plated at a density and under conditions where cell-cell contacts are very rare and the cells infrequently contract.

### siRNA transfection of NRVMs

A total of 20 μM stock siRNAs (diluted in DNase/RNase-free molecular-grade water) was diluted to a concentration of 120 nM using antibiotic-free DMEM/F:12 supplemented with 0.5% FCS v/v. Twenty-four hours after plating, the plating medium was aspirated and replaced with 1 ml of the resulting siRNA solutions composed of DMEM/F:12 supplemented with 0.5% FCS v/v and 0.675% (v/v) HiPerFect Transfection Reagent (Qiagen, Cat. No. 301704), which was further incubated for 15 min at room temperature prior to being added to the culture dishes. The cultures were then incubated at 37 °C in an incubator infused with 95% N_2_/5% CO_2_ for 7 h, followed by replacement of transfection media with DMEM/F:12 supplemented with penicillin/streptomycin and 0, 2, or 10% FCS where indicated. To prevent atrophy in myocytes maintained in 0% FCS, these cells were supplemented with ITS (24, 25). The ITS supplementation in the absence of FCS may be part of the reason we observe a lower amount of total protein in this condition.

### Treatment of NRVMs with PE

To stimulate growth *via* PE, NRVMs were first serum-starved for 48 h with serum-free DMEM/F:12 supplemented with penicillin/streptomycin and ITS, and then treated with 50 μM PE (Sigma-Aldrich, Cat. No. P6126) dissolved in DMEM/F:12 supplemented with ITS and antibiotics for 4 to 24 h (Fig. 5, *D* and *E*). After transfection with siRNA, NRVMs were first serum-starved for 48 h with serum-free DMEM/F:12 supplemented with penicillin/streptomycin and ITS, and then treated with 50 μM PE for 8 or 12 h (Figs. 5*G* and S3).

### siRNA sequences: siRNA oligonucleotides were purchased from integrated DNA technologies (IDT)

Negative Control DsiRNA (5 nmol, Cat. No. 51-01-14-04), a.k.a. “scrambled siRNA” is a nontargeting siRNA indicated by the manufacturer to not interact with any sequences in the human, mouse, or rat transcriptomes.

Bmal1 siRNA 1: 5′-rArGrUrArGrArArUrArCrArUrUrGrUrCrUrCrArArCrCrAAC-3′

Bmal1 siRNA 2: 5′-rCrArUrCrCrArArArArGrArUrArUrUrGrCrCrArArArGrUTA-3′

Sik1 siRNA 1: 5′-rGrCrUrArUrUrArArGrGrUrArCrUrArGrArArUrUrGrArUAA-3′

H3.3a siRNA: 5′-rArCrUrCrCrGrArGrArArArUrCrArGrArCrGrCrUrArUCA-3′

### Whole cell lysis

Medium was removed from 6-well culture dishes, and adherent cells were washed twice with 1 ml/well ice-cold Dulbecco's phosphate-buffered saline (DPBS) and then lysed with 40-70 μl of whole cell lysis buffer composed of 50 mM Tris (pH 8), 10 mM EDTA, 1% SDS, and 1× protease/phosphatase inhibitor mixture (Roche Applied Science; Catalog No. 05892791001 and 4906837001). Whole cell lysates were scraped and transferred to microcentrifuge tubes and stored at −80 °C. Lysates were subsequently thawed and briefly vortexed, followed by clarification of lysate *via* centrifugation at 20,000*g* for 5 min. Protein concentration of clarified cell lysates was measured *via* BCA protein assay kit (Thermo Fisher Scientific, Cat. No. J63283.QA) using bovine serum albumin (BSA) dissolved in water as a standard.

### Subnuclear fractionation

To isolate nuclei, NRVM cultures (5–6 wells per sample) were washed twice with 1 ml/well ice-cold DPBS and lysed with 100 μl of lysis buffer composed of 10 mM Tris (pH 7.4), 250 mM sucrose, 1 mM EDTA, 0.15% Nonidet P-40_subsitute_, and protease/phosphatase inhibitors, and scraped into Eppendorf tubes kept on ice. Samples were then centrifuged at 1000*g* for 10 min, yielding a crude nuclear pellet. The crude nuclear pellet was then resuspended in 100 μl and layered on top of a 300 μl sucrose cushion composed on 1.6 M sucrose, 15 mM NaCl, 10 mM Tris pH 7.4, and protease/phosphatase inhibitors. This was followed by centrifugation at 7500*g* for 10 min. The resulting nuclear pellet was then washed once with lysis buffer composed of 10 mM Tris (pH 7.4), 250 mM sucrose, 1 mM EDTA, 0.15% Nonidet P-40_subsitute_, and protease/phosphatase inhibitors and centrifuged at 7500*g* for 5 min. Chromatin was isolated from nuclei by resuspending nuclei in lysis buffer composed of 20 mM Hepes, 7.5 mM MgCl_2_, 30 mM NaCl, 1M urea, 1% Nonidet P-40_subsitute_, and protease/phosphatase inhibitors. This was followed by centrifugation at 13,000*g* for 15 min, resulting in a supernatant containing the nucleoplasm and solubilized nuclear membranes and a chromatin pellet. The chromatin pellet was then incubated at 4 °C for 16 h in 100 μl of 0.4 N H_2_SO_4_. Samples were then centrifuged at 15,000*g* for 10 min, and the supernatant was transferred to a new tube and mixed with 33 μl of 6.1 N trichloroacetic acid and kept on ice for 30 min to precipitate the acid-soluble chromatin fraction. This was followed by centrifugation at 20,000*g* for 20 min at 4 °C. The supernatant was discarded while the pellet was washed once with 250 μl ice-cold acetone and allowed to air dry. Pellets were then dissolved in 10-20 μl cell lysis buffer composed of 50 mM Tris (pH 8) 1% SDS, 1× protease/phosphatase inhibitor and quantified *via* bicinchoninic acid assay as described above.

### Covalent attachment of tags to capture histones and identify turnover over (CATCH-IT)

Fibroblasts isolated from P3 rat hearts were plated at 200,000 cells/well on 6-well dishes in DMEM/F:12 supplemented with 10% FCS and antibiotics and incubated overnight (∼16 h). Cultures were then switched to serum-free DMEM/F:12 supplemented with ITS and antibiotics for 72 h to halt cell cycling, and then switched to DMEM/F:12 supplemented with 20% FCS and antibiotics to stimulate synchronous cell cycling (36). Cultures were then treated with L-azidohomoalanine hydrochloride (AHA) (Sigma-Aldrich, Cat. No. 900892) at a concentration of 4 mM at 5 h intervals after switching media to 20%. Cultures were then subjected to acid extraction of histones as described above. Subsequently, 10 to 30 μg of acid-soluble chromatin fraction were then precleared with 50 μl of preequilibrated streptavidin M-280 Dynabeads (Thermo Fisher Scientific, Cat. No. 11205D), and then subjected to click chemistry by adding the following reagents to the stated final concentrations: 300 μM Tris(3-hydroxypropyltriazolylmethyl)amine (THPTA) (Sigma-Aldrich, Cat. No. 762342), 750 μM Ascorbic acid (Sigma-Aldrich, Cat.No. AX1775), 400 μM CuSO_4_ (Sigma-Aldrich, Cat. No. C1297), and 7.5 μM Biotin-PEG4-alkyne (Sigma-Aldrich, Cat. No. 764213). The reactions were incubated at room temperature for 1.5 h and then precipitated with trichloroacetic acid as described above to remove excess click chemistry reagents. Precipitated protein was resuspended in 1% SDS, 15 mM NaCl, and 10 mM Tris pH 7.4, and incubated with 100 μl of preequilibrated streptavidin M280 Dynabeads at room temperature for 1.5 h with agitation every 15 min. The beads were washed four times with 500 μl of lysis buffer containing 1% SDS, 15 mM NaCl, and 10 mM Tris pH 7.4. To elute bound biotinylated proteins, beads were incubated at 100 °C for 5 min in 30 μl 1× NuPAGE LDS sample buffer (Thermo Fisher Scientific, Cat. No. NP0007) supplemented with 25 mM Biotin (PMID: 28986262) and 1.25% v/v β-mercaptoethanol. Eluted proteins were then resolved *via* SDS-PAGE on 15% polyacrylamide gels and immunoblotted for histone H3 as described below.

In Figure 3, NRVMs transfected with control or Bmal1-targeted siRNA as described above were maintained in serum-free medium supplemented with ITS for 72 h. Following medium aspiration, cultures were treated with serum-free medium supplemented with ITS and 4 mM AHA (1 ml/well) and incubated at 37 °C for 4 h. In Fig. S4, NRVMs were first serum-starved for 48 h with serum-free DMEM/F:12 supplemented with penicillin/streptomycin and ITS, and then treated with 50 μM PE dissolved in DMEM/F:12 supplemented with ITS and antibiotics for 12 h, and labeled with 4 mM AHA at hours 8 to 12 of PE treatment. After the isolation of acid-soluble chromatin fractions as described above, click reactions with Biotin-PEG4-alkyne were performed as described above using 250 to 1000 ng of protein, followed by SDS-PAGE and blotting with Streptavidin-HRP (Jackson ImmunoResearch, Cat. No. 016-030-084).

### SDS-PAGE

SDS-PAGE electrophoresis buffer was composed of 24.2 g Tris base, 115.2 g glycine, 8 g SDS, filled to a final volume of 8 l. Immunoblot transfer buffer was composed of 24.2 g Tris base, 115.2 g glycine, 1.28 l methanol, filled to a final volume of 8 l. SDS-PAGE gels were cast using mini-PROTEAN spacer plates with 1.5 mm integrated spacers (Bio-Rad Cat. No. 1653312), mini-PROTEAN short plates (Bio-Rad, Cat. No. 1653308), and 15-well 1.5 mm Mini-PROTEAN combs (Bio-Rad, Cat. No. 1653366).

### Total protein staining

Where indicated, after SDS-PAGE of 1-2 ug of whole cell lysate or 500-1000 ng of acid-soluble chromatin fractions isolated from NRVMs, gels were stained with Oriole UV-fluorescent stain per the manufacturer’s instructions (Bio-Rad Cat. No. #1610496) to visualize and quantify total protein loading.

### Bmal1, GAPDH, and cyclin A2 immunoblots

Around 5 to 7 μg of whole cell protein lysates were subjected to SDS-PAGE at 200 V for approximately 1 h using 15% polyacrylamide gels. This was followed by electroelution onto polyvinylidene fluoride membranes at 100 V for 50 min under semidry transfer conditions at 4 °C.

### Histone H3, H4, H2B, and H2A immunoblots

In addition, 1 μg of whole cell protein lysates or 50 to 200 ng of acid-soluble chromatin fractions were subjected to SDS-PAGE at 200 V for 50 min on 15% polyacrylamide gels. This was followed by electroelution onto polyvinylidene fluoride membranes at 100 V for 50 min under semidry transfer conditions at 4 °C. After electroelution, membranes were placed in methanol for 30 s and placed on paper towels to allow methanol to evaporate from the membranes. Membranes were then again placed in methanol for 30 s with gentle rocking, followed by placement into molecular-grade water with gentle rocking again for 30 s, followed by 12 to 16 h at 4 °C in PBS supplemented with 0.01% Tween-20 (Sigma-Aldrich, Cat No. A7030) (PBST), 5% BSA (Cat. No. P2287), and the appropriate antibody. Membranes were then washed three times for 15 min in PBST and then incubated for 45 min at room temperature with the appropriate HRP-conjugated anti-IgG (Jackson ImmunoResearch Laboratories, Inc) diluted at 1:2000 in 5% BSA dissolved in PBST. Membranes were then washed three times for 15 min with gentle rocking in PBST, subjected to enhanced chemiluminescence imaged using a Bio-Rad ChemiDoc System. Immunoblots were quantified using ImageJ software (https://imagej.net/ij/download.html) densitometry.

### Antibodies used in this study

Bmal1 (Abcam: ab93806); GAPDH (EMD Millipore-Sigma: MAB374); histone H3 (Cell Signaling Technology: 9715); histone H2B (Abcam: ab1790); histone H4 (Abcam: ab10158); histone H2A (Abcam: ab18255); and cyclin 2A (Abcam: ab181591); H3.3 (Abcam: ab176840).

### mRNA isolation

Medium was removed from 6-well culture dishes, and adherent cells were washed with ice-cold DPBS and then lysed with 300 μl of TRIzol Reagent (Thermo Fisher Scientific, Cat. No. 15596026). The resulting lysates were homogenized and scraped into Eppendorf tubes and stored at −80 °C. After thawing samples at room temperature (∼20 °C), 60 μl of chloroform was added to each sample and shaken vigorously by hand for 15 s. Samples were then incubated at room temperature for 2 to 3 min to allow separation of layers. The samples were then centrifuged at 13,000 rpm for 15 min at 4 °C. The resulting upper clear layer (approximately 150 μl) from each sample was pipetted into a fresh Eppendorf tube, and 150 μl of isopropanol was added to each sample, followed by mixing *via* ten inversions. The samples were then centrifuged at 13,000 rpm for 20 min at 4 °C. At this point, the samples are kept on ice, and the supernatant was removed without disturbing the visible pellet. One milliliter of 75% reagent-grade ethanol was then added to each tube, followed by centrifugation at 15,000 rpm for 5 min at 4 °C. The supernatant was discarded, followed by centrifugation at 15,000 rpm for 5 min at 4 °C, and any residual supernatant was also removed and discarded. The Eppendorf tubes were then opened and placed on a heat block set to 55 °C for 5 to 10 min (or until no liquid remains). Subsequently, 10 μl of DNase/RNase-free water was then added to the bottom of each tube, and the tubes were then closed and placed back on the heating block for 5 min to dissolve the RNA. This was then followed by centrifugation at 15,000 rpm for 5 min at 4 °C. The RNA concentration of the samples was then quantified by nanodrop.

### cDNA synthesis

After the quantification of mRNA concentration, 30 to 500 ng of mRNA per sample was used to generate complementary DNA (cDNA) using an iScript cDNA Synthesis Kit (20 μl reaction volume) using the following reaction protocol: priming: 5 min at 25 °C; reverse transcription: 50 min at 46 °C; reverse transcription inactivation: 1 min at 95 °C. After cDNA synthesis, the cDNA synthesis reactions were diluted with RNase/DNase free water, for example, for every 500 ng of mRNA used, 80 μl of water was added to the cDNA reaction.

### RT-qPCR

Following dilution of cDNA reactions with water, target cDNA amplification was measured using the following reaction components: 5 μl diluted cDNA, 1 μl of 5 μM forward primer stock, 1 μl of 5 μM reverse primer stock, 3 μl of RNase/DNase free water, and 10 μl SsoFast EvaGreen Supermix (Bio-Rad, Cat. No. 1725201).

### MNase-qPCR

Medium was removed from 6-well culture dishes, and adherent cells were washed with ice-cold DPBS and then lysed with 100 μl of ice-cold lysis buffer composed of 10 mM Tris–HCl, pH 7.4, 10 mM NaCl, 2 mM MgCl_2_, 0.5% Nonidet P-40_Substitute_ and 1× protease/phosphatase inhibitor mixture. Lysates/intact nuclei were scraped and transferred to microcentrifuge tubes. For this specific protocol, each sample was composed of two wells scraped into the same tube. The resulting crude nuclear suspensions were then centrifuged at 1000*g* for 10 min. At this point, the samples were maintained on ice. The supernatant was discarded, and the crude nuclear pellet was then gently but thoroughly resuspended in 120 μl of prewarmed MNase reaction buffer, followed by addition of 120 μl of prewarmed MNase reaction buffer (composed of 10 mM Tris, pH 7.4, 5 mM NaCl, and 1 mM CaCl_2_•2H_2_O) containing 0U, 0.001U, 0.01U, or 0.1U MNase (MNase, Worthington, LS004798). The reaction vessels were then immediately placed in a 37 ºC water bath for 5 min. This was followed by addition of 99 μl of MNase stop reaction buffer composed of 60 μL 100 mM EDTA, 10 mM EGTA, 30 μl 20% SDS, and 9 μl of 25 mg/ml Proteinase K. The resulting solutions were then incubated for 16 h at 37 °C. Afterward, 340 μl of phenol:chloroform:isoamyl alcohol (25:24:1) was then added to each tube and gently shaken by hand for 20 s. Samples were then centrifuged at 16,000*g* at room temperature for 5 min. The upper aqueous layer was then transferred to a 2 ml Eppendorf tube. To each tube, the following were added in the stated order: 4.25 μl of 15 mg/ml Glycoblue (Thermo Fisher Scientific, Cat. No. AM9515), 170 μl 7.5 M ammonium acetate, and 1.27 ml 100% reagent-grade ethanol. Samples were then stored at −80 °C for 1 h. The samples were then centrifuged at 16,000*g* for 30 min at 4 °C to pellet DNA. The supernatant was discarded, and 150 μl of 70% reagent-grade ethanol was added, followed by centrifugation at 16,000*g* for 2 min at 4 °C. The supernatant was discarded, and another 150 μl of 70% reagent-grade ethanol was added, followed by centrifugation at 16,000*g* for 2 min at 4 °C. The supernatant was discarded, and the DNA pellets were allowed to air dry, followed by resuspension in 20 μl DNase/RNase-free water and quantification by nanodrop. Subsequently, 100 ng of DNA samples were resolved *via* electrophoresis on a 1% agarose gel in 1× Tris acetic acid EDTA buffer, 120 V, 1 h at room temperature (21 °C). Chromatin accessibility was then assessed *via* RT-qPCR using the following reaction components: 5 μl diluted genomic DNA (60 ng/reaction), 1 μl of a 5 μM of a forward primer stock, 1 μl of a 5 μM reverse primer stock, 3 μl of RNase/DNase free water, and 10 μl SsoFast EvaGreen Supermix (39).

### Cell size measurements

NRVM cell size was measured *via* ImageJ. To accurately measure cell size in μm^2^, an image of a hemocytometer (Thermo Fisher Scientific, Cat. No. 02-671-6) was used to set the scale in ImageJ, and the individual cells were manually traced on a Lenovo T460 Thinkpad with touchscreen.

### g:Profiler analyses

RNA-Seq data generated from adult and embryonic mouse myocytes in a previous study were downloaded into a new excel file and filtered for all genes significantly upregulated in adult myocytes (*p* < 0.05). The resulting gene list was then filtered for genes containing Bmal1 ChIP-seq peaks in their promoters/TSS found across organs, or for genes containing Bmal1 ChIP-seq peaks in their promoters/TSS specific to the heart. The resulting gene lists were then subjected to gene ontology analysis *via* g:Profiler analysis using default parameters (46).

### IGV browser track viewing

The BW files (6, 47) or bedgraph files (38) shown in Figure 5 were downloaded from NCBI gene expression omnibus and displayed on Integrated Genomics Viewer (IGV).

## Data availability

All primary data are available upon request from Dr Thomas Vondriska (tvondriska@mednet.ucla.edu). Previously published and publicly available data are accessible from GEO using accession numbers GSE110604 (21), GSE124008 (35), and GSE102532 (38).

## Supporting information

This article contains supporting information.

## Conflict of interest

The authors declare that they have no conflicts of interest with the contents of this article.
