# Supplementary material for: Circadian control of histone turnover during cardiac development and growth
Source: J Biol Chem. 2024 Jun 1;300(7):107434. doi: 10.1016/j.jbc.2024.107434 (PMC11261805; doi:10.1016/j.jbc.2024.107434)
Supplement: Supplemental Figures S1–S4 [file mmc1.pdf]

**Figure S1**

**A.** Culture NRVM in 10% FCS (24Hr) → Transfect with Control/Bmal1 siRNA in 0.5% FCS (7Hr) **t=0 at time of siRNA addition** → After 7Hr transfection Maintain in 0, 2, or 10% FCS → Image, collect whole cell lysate or RNA.

**B.**

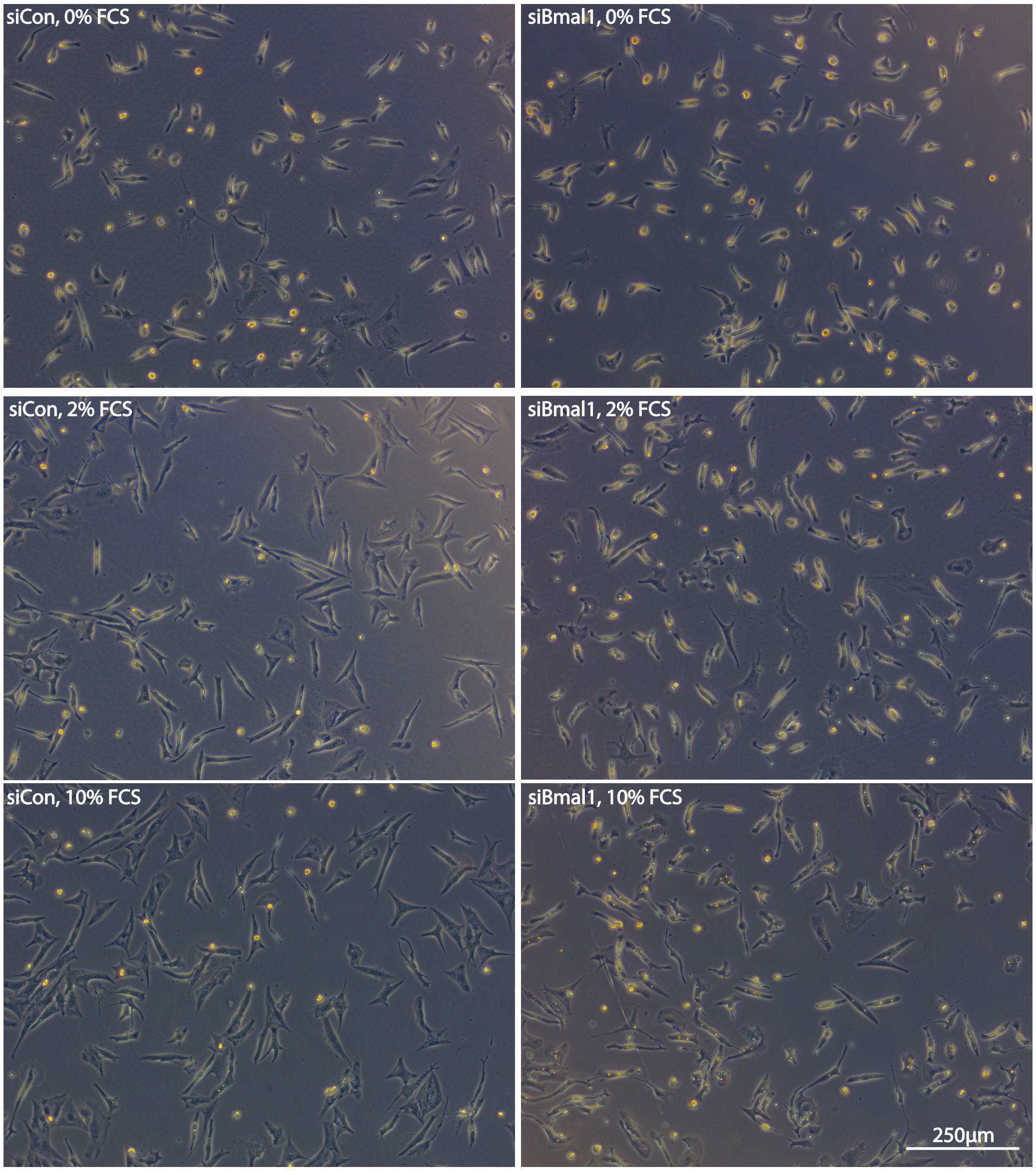

**Figure S2**

**A.**

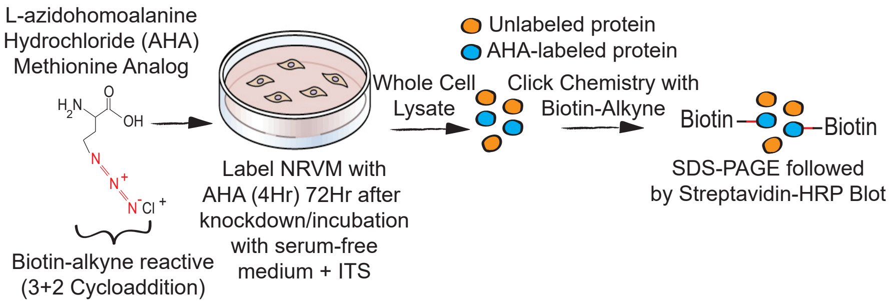

L-azidohomoalanine
Hydrochloride (AHA)
Methionine Analog
Biotin-alkyne reactive
(3+2 Cycloaddition)
Label NRVM with
AHA (4Hr) 72Hr after
knockdown/incubation
with serum-free
medium + ITS
Whole Cell
Lysate
Unlabeled protein
AHA-labeled protein
Click Chemistry with
Biotin-Alkyne
Biotin
Biotin
SDS-PAGE followed
by Streptavidin-HRP Blot


**B.**

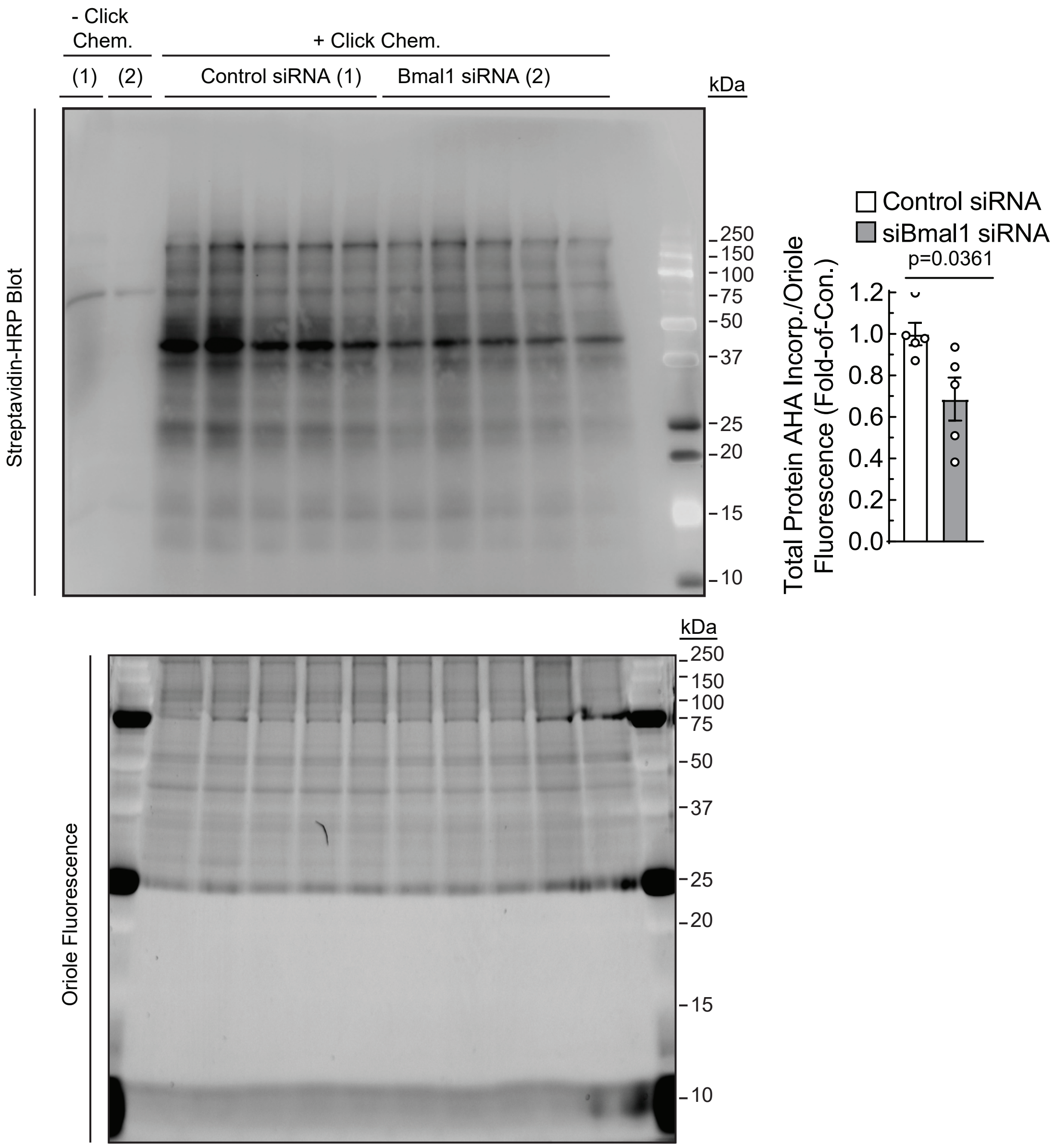

- Click
Chem.
+ Click Chem.
(1)
(2)
Control siRNA (1)
Bmal1 siRNA (2)
kDa
Streptavidin-HRP Blot
250
150
100
75
50
37
25
20
15
10
Control siRNA
siBmal1 siRNA
p=0.0361
Total Protein AHA Incorp./Oriole
Fluorescence (Fold-of-Con.)
1.2
1.0
0.8
0.6
0.4
0.2
0.0
kDa
250
150
100
75
50
37
25
20
15
10
Oriole Fluorescence

# Figure S3

## A.

Culture NRVM in 10% FCS (24Hr) ➔ Transfect with Control/Bmal1 siRNA, **t=0 at time of siRNA addition** ➔ After 7Hr transfection maintain in serum-free medium for 48Hr ➔ Treat with 50um PE, 12Hr in serum-free medium ➔ For 8-12Hr of PE treatment, label with 4mM AHA, isolate and acid extract chromatin: biotinylate metabolically labeled proteins via click reaction, blot with Streptavidin-HRP.

## B.

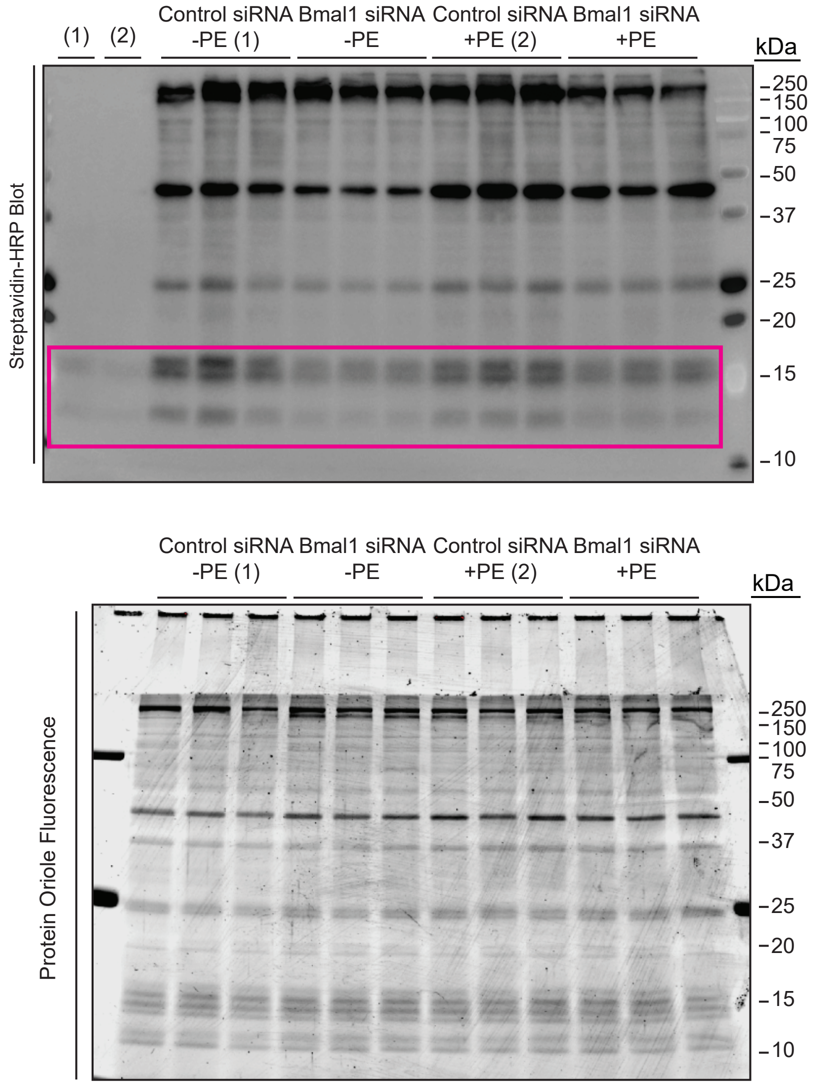


## C.

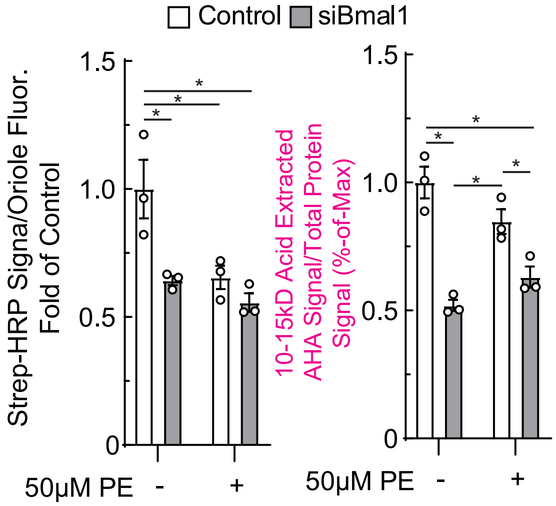

## Figure S4

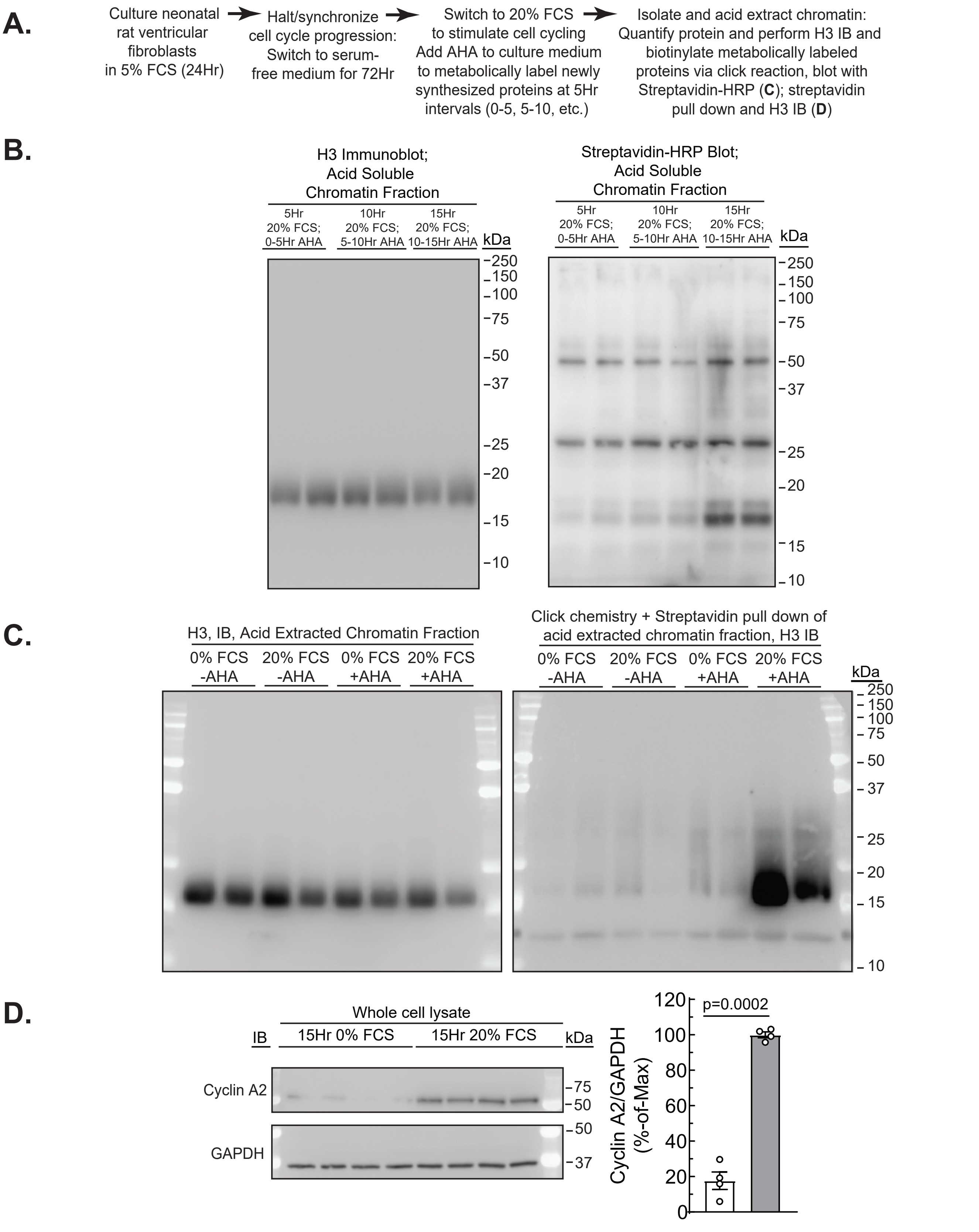

A.
Culture neonatal rat ventricular fibroblasts in 5% FCS (24Hr)
Halt/synchronize cell cycle progression: Switch to serum-free medium for 72Hr
Switch to 20% FCS to stimulate cell cycling Add AHA to culture medium to metabolically label newly synthesized proteins at 5Hr intervals (0-5, 5-10, etc.)
Isolate and acid extract chromatin: Quantify protein and perform H3 IB and biotinylate metabolically labeled proteins via click reaction, blot with Streptavidin-HRP (C); streptavidin pull down and H3 IB (D)
B.
H3 Immunoblot; Acid Soluble Chromatin Fraction
5Hr 20% FCS; 0-5Hr AHA
10Hr 20% FCS; 5-10Hr AHA
15Hr 20% FCS; 10-15Hr AHA
kDa
250
150
100
75
50
37
25
20
15
10
Streptavidin-HRP Blot; Acid Soluble Chromatin Fraction
5Hr 20% FCS; 0-5Hr AHA
10Hr 20% FCS; 5-10Hr AHA
15Hr 20% FCS; 10-15Hr AHA
kDa
250
150
100
75
50
37
25
20
15
10
C.
H3, IB, Acid Extracted Chromatin Fraction
0% FCS -AHA
20% FCS -AHA
0% FCS +AHA
20% FCS +AHA
Click chemistry + Streptavidin pull down of acid extracted chromatin fraction, H3 IB
0% FCS -AHA
20% FCS -AHA
0% FCS +AHA
20% FCS +AHA
kDa
250
150
100
75
50
37
25
20
15
10
D.
Whole cell lysate
IB
15Hr 0% FCS
15Hr 20% FCS
kDa
Cyclin A2
75
50
GAPDH
50
37
Cyclin A2/GAPDH (%-of-Max)
p=0.0002
120
100
80
60
40
20
0
