# Supplementary material for: Circadian control of histone turnover during cardiac development and growth
Source: J Biol Chem. 2024 Jun 1;300(7):107434. doi: 10.1016/j.jbc.2024.107434 (PMC11261805; doi:10.1016/j.jbc.2024.107434)
Supplement: Supplemental Table [file mmc2.docx]

**Reverse Transcription RT-qPCR primers:**

| Target | Sense (5’-3’) | Antisense (5’-3’) |
| --- | --- | --- |
| Bmal1 | ATTGACGAATTGGCTTCTTTGG | CCTGAGCACGGTGAGTTTAT |
| Gapdh | CCTGGCCAAGGTCATCCAT | GTCATGAGCCCTTCCACGAT |
| Nppb | GTGCTGCCCCAGATGATTCT | CAGCGGCGACAGATTAAGGA |
| Nppb_Intron_ | ATTAACCACGCGAGAGTCAG | CCTGTCGGTTAAGGGATCTG |
| Per2 | AAAGGAGCTGCGGATGTTAG | GTCATCATGAGTCTGAAGGTATCG |
| Per2_Intron_ | CTCCCAGTTCCCAGTACTCA | CTTGGTGCCTAGCTGTAGTTG |
| Sik1 | GGCATACACTGGCTGAAGT | GGCAGAGGAGGAGACAATTATG |
| Sik1_Intron_ | GGATCCGAGCCTTGATGTTTA | GCTTAATCTAGGCCACCAGAG |
| Tcap | GATGTAGAATGCTCCGGAAGG | GTGCTAACAGAGGGTGCTG |
| Tcap_Intron_ | CGAGGAACAGAAGGATGAGAAG | TGGTTCTCCTTGAGTCTTCTTG |

**MNase-qPCR primers:**

| Target | Sense (5’-3’) | Antisense (5’-3’) |
| --- | --- | --- |
| Per2 | GGAAGTGGACGAGCCTACTC | ACATAAGACGCACATGGAACTC |
| Sik1_Proximal_ | CCTCCAGTCTCTTGGCAACC | ACAGCCCATTGACGTCGTTT |
| Sik1_Distal_ | CAGCCACCCTGTGTCCTATC | GCACTTTGCCCATTCCCTTG |
