# Supplementary material for: Circadian control of histone turnover during cardiac development and growth
Source: J Biol Chem. 2024 Jun 1;300(7):107434. doi: 10.1016/j.jbc.2024.107434 (PMC11261805; doi:10.1016/j.jbc.2024.107434)
Supplement: Supplemental Figures S1–S4 Legend [file mmc3.docx]

**Supplementary Figure Legends**

**Supplementary Figure 1**: **A**) Experimental timeline of NRVM treatment with serum and Bmal1 knockdown. B) Brightfield images from which cell size was quantified for Fig. 1E.

**Supplementary Figure 2: Effect of Bmal1 knockdown on AHA incorporation into total protein. A**) Schematic of L-azidohomoalanine (AHA, methionine analog) labeling in NRVMs. After labeling with 4mM AHA for 4Hr, protein lysates containing newly synthesized AHA-labeled proteins are alkylated via click chemistry with alkyne-biotin and visualized with streptavidin-HRP enhanced chemiluminescence. **B**) Streptavidin-HRP blot of AHA-labeled protein extracts from NRVM transfected with control or Bmal1-targeting siRNA, and quantification of streptavidin-HRP signal normalized to oriole fluorescence signal (p-value from unpaired t-test; mean ± SEM).

**Supplementary Figure 3**: **Effect of PE on histone turnover in NRVM.** **A**) Experimental timeline of NRVM treated PE and AHA labeling performed during hours 8-12 of PE treatment, followed by acid extraction of chromatin fractions and click chemistry with Biotin-Alkyne. **B**) *Top*, Streptavidin-HRP blot and *bottom*, Oriole total protein stain (quantified in **C**, whole blot on left, boxed area normalized to entire blot on right; * indicates p<0.05 by two-way ANOVA with Tukey’s post hoc analysis for pairwise comparisons; mean ± SEM).

**Supplementary Figure 4: AHA incorporation into total histone H3 in cell cycle synchronized neonatal rat ventricular fibroblasts. A**) Experimental timeline of rat fibroblasts subjected to cell cycle synchronization in combination with AHA labeling at different intervals after serum treatment, followed by acid extraction of chromatin-associated histones and subsequent detection of AHA-labeled proteins with alkyne-biotin and Streptavidin-HRP. **B**) Histone H3 immunoblot (left) and Streptavidin-HRP blot of acid soluble chromatin fractions from fibroblasts treated with serum and 4mM AHA for the indicated times. **C**) Histone H3 immunoblots of acid extracted chromatin fractions and concomitant streptavidin pulldowns of AHA-labeled proteins from fibroblasts treated with or without 20% FCS for 15Hr and with or without 4mM AHA for the last 5Hr. **D**) Cyclin A2 and GAPDH immunoblots indicating increased AHA labeling observed in **B** and **C** occurs during S-phase of the cell cycle (p-values from unpaired t-test; mean ± SEM).
